# Simplified, Enhanced Protein Purification Using an Inducible, Autoprocessing Enzyme Tag

**DOI:** 10.1371/journal.pone.0008119

**Published:** 2009-12-02

**Authors:** Aimee Shen, Patrick J. Lupardus, Montse Morell, Elizabeth L. Ponder, A. Masoud Sadaghiani, K. Christopher Garcia, Matthew Bogyo

**Affiliations:** 1 Department of Pathology, Stanford School of Medicine, Stanford, California, United States of America; 2 Department of Molecular and Cellular Physiology, Stanford School of Medicine, Stanford, California, United States of America; 3 Department of Systems and Chemical Biology, Stanford School of Medicine, Stanford, California, United States of America; 4 Howard Hughes Institute, Stanford School of Medicine, Stanford, California, United States of America; University of Washington, United States of America

## Abstract

We introduce a new method for purifying recombinant proteins expressed in bacteria using a highly specific, inducible, self-cleaving protease tag. This tag is comprised of the *Vibrio cholerae* MARTX toxin cysteine protease domain (CPD), an autoprocessing enzyme that cleaves exclusively after a leucine residue within the target protein-CPD junction. Importantly, *V. cholerae* CPD is specifically activated by inositol hexakisphosphate (InsP_6_), a eukaryotic-specific small molecule that is absent from the bacterial cytosol. As a result, when His_6_-tagged CPD is fused to the C-terminus of target proteins and expressed in *Escherichia coli*, the full-length fusion protein can be purified from bacterial lysates using metal ion affinity chromatography. Subsequent addition of InsP_6_ to the immobilized fusion protein induces CPD-mediated cleavage at the target protein-CPD junction, releasing untagged target protein into the supernatant. This method condenses affinity chromatography and fusion tag cleavage into a single step, obviating the need for exogenous protease addition to remove the fusion tag(s) and increasing the efficiency of tag separation. Furthermore, in addition to being timesaving, versatile, and inexpensive, our results indicate that the CPD purification system can enhance the expression, integrity, and solubility of intractable proteins from diverse organisms.

## Introduction

The availability of simple, reliable, and cost-effective methods for recombinant protein purification is critical for the work of high throughput structural and proteomic centers and many individual researchers alike. While the addition of affinity tags such as poly-His and glutathione transferase (GST) to target proteins has greatly simplified purification strategies, it is often difficult to obtain soluble recombinant protein [1]. As a result, intractable affinity-tagged target proteins are often fused to small proteins such as NusA and SUMO to improve their solubility, expression, and stability [2].

Since these tags can alter the biological activity of target proteins and interfere with protein crystallization studies, many biological and biomedical applications require that the tag be removed from the target protein. Most commonly used methods involve the addition of exogenous site-specific proteases to cleave the affinity tag off the target protein at engineered sites [2]. Unfortunately, high levels of endoprotease must often be applied for extended periods of time, and this can result in undesirable cleavages within the target protein. Furthermore, these endoproteases are costly, often exhibit poor solubility, and require the inclusion of additional chromatography steps to remove the exogenous protease.

**Figure 1:**
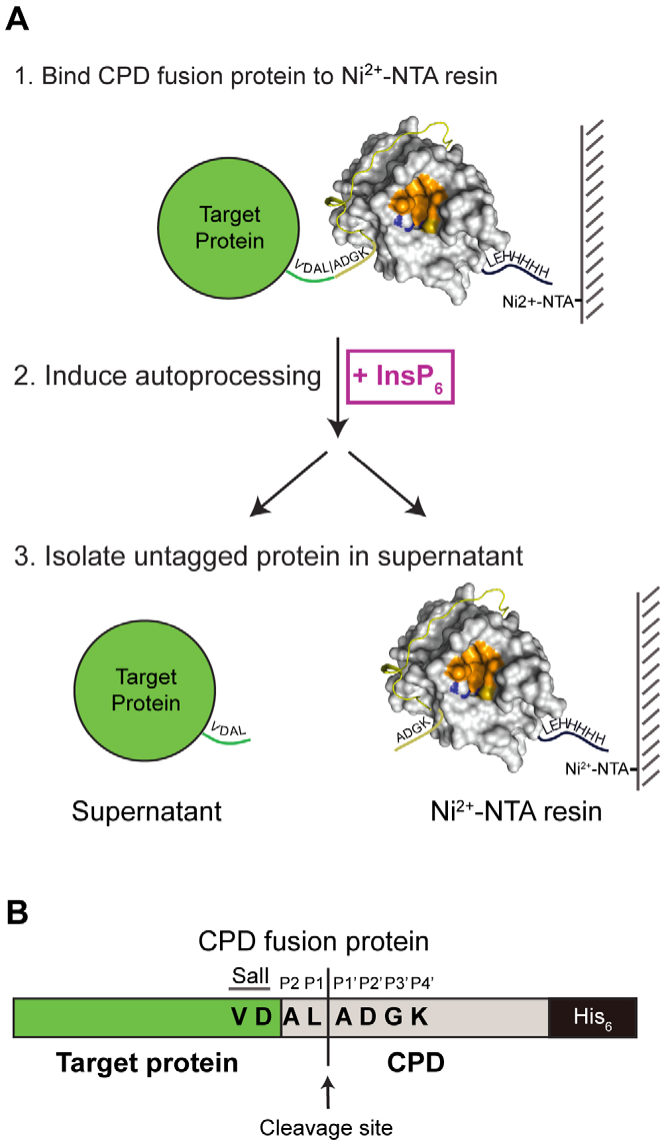
CPD fusion protein purification system. (A) Schematic of target protein purification using the CPD, described in detail in the text. (B) Schematic of CPD fusion protein. The P4 and P3 residues Val and Asp, respectively, encoded by the SalI site, and the remaining P2-P4′ residues contained within the CPD are shown. Prime positions refer to residues C-terminal to the autocleavage site, which is demarcated as a black vertical line. The composition of residues appended to the C-terminus of target proteins following autoprocessing can vary between one and four residues as described in Figure 2. At present, the CPD system functions as a C-terminal fusion to target proteins and thus complements existing methods in which the affinity tag can only be applied as an N-terminal fusion [18].

To circumvent these disadvantages, we have developed an on-bead cleavage purification system in which a site-specific affinity-tagged protease is fused directly to the target protein. This approach condenses affinity purification, cleavage, and tag separation into a single step, simplifying protein purification procedures and increasing purification yields. The key element of this purification method is the *Vibrio cholerae* MARTX toxin cysteine protease domain (CPD) [3]. The CPD exhibits several properties that facilitate its development into an inducible, autocleaving protease tag. First, the CPD is a highly specific protease that cleaves exclusively after Leu residues [4]. Second, the CPD is inducible, as it is specifically activated by the eukaryotic-specific small molecule inositol hexakisphosphate (InsP_6_) [5], [6]. Since InsP_6_ is absent from bacterial cells [7], [8], full-length CPD-His_6_ fusion proteins can be purified from bacterial lysates in a protease-inactive form using imidzaole affinity chromatography (IMAC). Addition of InsP_6_ to an immobilized, C-terminally His_6_-tagged fusion protein induces autoprocessing at the P1 Leu cleavage site (P1 refers to the residue N-terminal to the scissile bond), which is located at the target protein-CPD junction (Figure 1). This processing event releases the untagged target protein into the supernatant, while the C-terminally His_6_-tagged CPD remains immobilized on the Ni^2+^-NTA resin. Third, as an autoprocessing enzyme, the CPD exhibits poor transcleavage efficiency [4], [5]. This property should limit fusion protein cleavage to the CPD-target protein junction and permit the high fidelity removal of the His_6_-CPD tag from the target protein.

In this report, we demonstrate using a variety of target proteins that this novel purification system combines the simplicity of one-step purification systems [9], [10] with many of the advantages of affinity tags [2] in that it can increase the expression, solubility, and integrity of target proteins. Thus, this method facilitates the rapid purification of both soluble and intractable, recombinant, untagged proteins, suggesting that it will have widespread utility in individual research labs and high-throughput structural and proteomic centers.

## Results

### Development of the One-Step CPD Purification System

**Figure 2:**
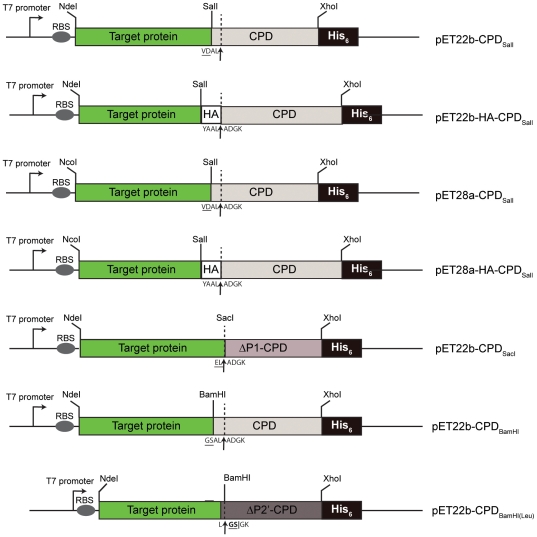
Schematic of pET-CPD expression vectors. Bent arrow, T7 promoter, Oval (RBS), ribosome binding site, green rectangle, target protein, grey rectangle, CPD, *V. cholerae* MARTX (aa. 3440–3650), darker grey rectangle, ΔP1-CPD, *V. cholerae* MARTX (aa. 3442–3650), darkest grey rectangle, ΔP2′-CPD, *V. cholerae* MARTX (aa. 3444–3650), black rectangle, His_6_-tag, white rectangle, HA-tag. The dotted vertical line and arrow indicate the CPD cleavage site. Residues added onto the C-terminus of the target protein following CPD-mediated cleavage, and the relevant restriction sites are shown (residues encoded by the restriction sites that are appended to the C-terminus of target proteins are underlined). The composition of the residues added to the C-terminus of the target protein can be varied depending on the cloning site and pET-CPD vector used. It should be noted that the P1 Leu shown for pET22b-CPD_BamHI-Leu_ must be encoded in the 3′ cloning primer of the target gene (i.e. add a Leu codon to the end of the target insert). Both pET22b and pET28a vector backbones were used to construct the CPD expression vectors.

In order to produce CPD fusion proteins, we first constructed CPD expression vectors (pET-CPD expression vectors) using the pET expression vector backbone. DNA encoding the CPD was cloned into the SalI restriction site (Figure 2) such that the fusion protein produced upon IPTG induction of *E. coli* harboring the pET-CPD_SalI_ vector carries the P2-P1 residues of the native CPD (Ala-Leu, respectively) and the P4-P3 residues encoded by the SalI site (Val-Asp, respectively) (Figures 1 and 2). The P1 residue refers to the amino acid N-terminal to the scissile bond, while the residue N-terminally adjacent to the P1 residue is termed P2, and so on. When InsP_6_ is added to induce CPD-mediated autocleavage of the fusion protein, the untagged target protein is released from the resin and carries four additional C-terminal residues (Val-Asp-Ala-Leu); the His_6_-tagged CPD remains bound to the resin (Figure 1). The Val-Asp-Ala-Leu C-terminal addition can be reduced to two amino acids (Glu-Leu) by cloning into the SacI site, or to a single amino acid (Leu) by cloning into the BamHI site and adding a Leu codon to the 3′ cloning primer (Figure 2).

**Figure 3:**
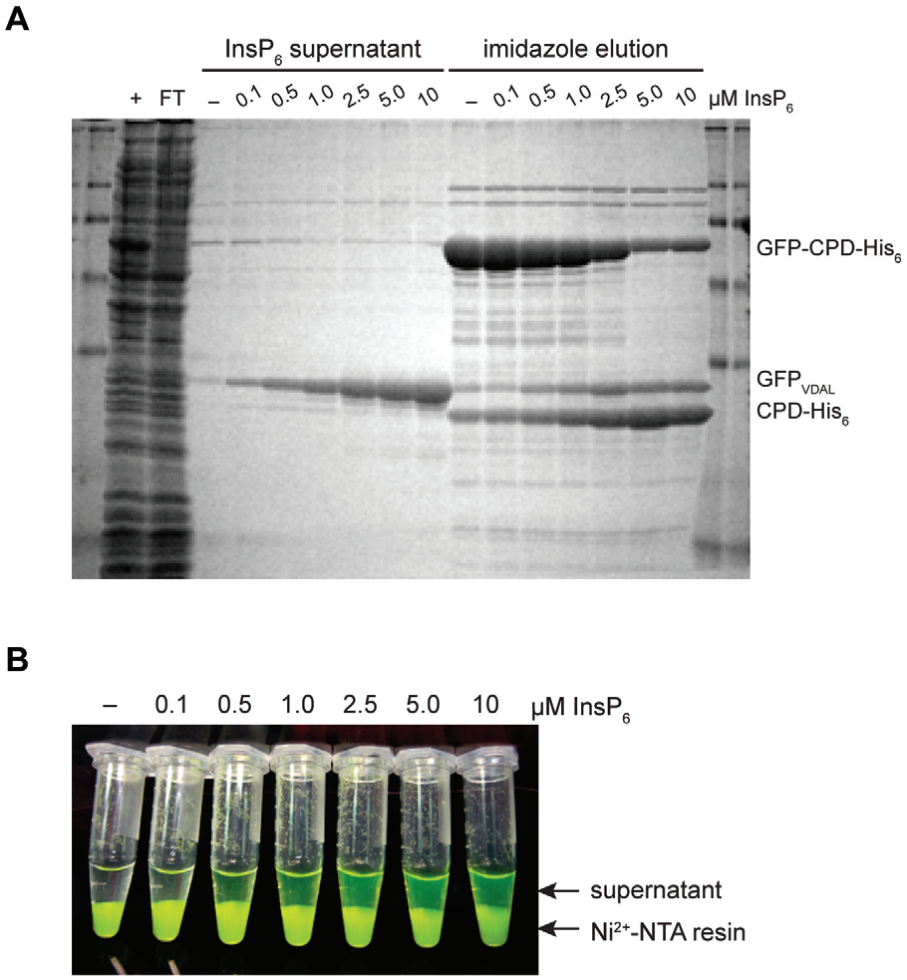
Purification of GFP using the CPD-His_6_ tag. (A) SDS-PAGE analysis of GFP purification using Coomassie stain. GFP-CPD-His_6_ bound to Ni^2+^-NTA resin was incubated with increasing amounts of InsP_6_ for 2 hrs at 4°C. GFP released into the supernatant was collected (InsP_6_ supernatant); Ni^2+^-bound proteins were then eluted from the resin by the addition of 200 mM imidazole (Imidazole elution). Collected fractions were analyzed by SDS-PAGE. (B) Visual analysis of GFP released into the supernatant fraction upon InsP_6_ addition to immobilized GFP-CPD-His_6_ fusion protein.

To demonstrate the feasibility of this system, we first expressed and purified green fluorescent protein (GFP) as a fusion to CPD-His_6_ using IMAC. As anticipated, addition of increasing amounts of InsP_6_ stimulated the release of GFP from the Ni^2+^-NTA resin in a dose-dependent manner (Figures 3A and B), while the His_6_-tagged CPD remained bound to the Ni^2+^-NTA agarose beads (bead eluate, Figure 3A).

### The CPD Cleaves Exclusively at the Fusion Protein Junction

**Figure 4 pone-0008119-g004:**
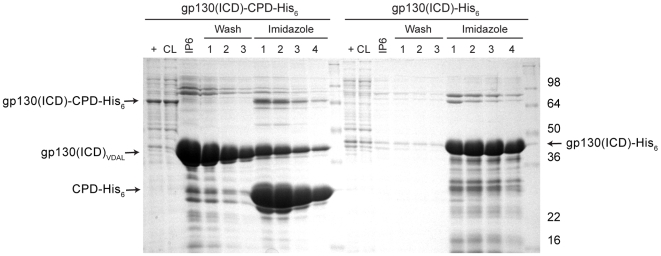
The CPD does not cleave within an intrinsically unstructured protein. gp130 intracellular domain (ICD)-CPD-His_6_ or gp130(ICD)-His_6_ bound to Ni^2+^-NTA resin was incubated with 100 µM InsP_6_ for 2 hr at room temperature; the resin was washed four times, followed by elution of Ni^2+^-bound proteins by 200 mM imidazole. Purification fractions were analyzed by SDS-PAGE followed by Coomassie staining. CL, cleared lysate, FT, flowthrough, IP6, elution from InsP_6_ incubation.

We have previously shown that *V. cholerae* CPD is positioned to undergo autocleavage at a proximal N-terminal leucine and that it exhibits significantly reduced transcleavage efficiency [4], [5], which should limit its ability to cleave target proteins at heterologous sites. Indeed, mutation of the P1 Leu to an Ile residue is sufficient to prevent CPD-mediated transcleavage, a finding that is explained by the observation that the P1 Leu residue fits snugly into the S1 substrate binding pocket in the crystal structure of the P1 Leu aza-epoxide inhibitor modified *V. cholerae* CPD [4]. Nevertheless, since other site-specific proteases used to remove fusion tags have been observed to cleave target proteins at secondary sites [2], we examined whether the CPD would spuriously cleave target proteins. Specifically, we tested whether the CPD would cleave an intrinsically disordered protein after Leu residues within the target protein. We used the intracellular domain (ICD) of the cytokine receptor gp130 as a test substrate, since it is unstructured in solution by NMR [11] and contains multiple Leu residues that might serve as cleavage substrates [12]. The ICD-CPD-His_6_ fusion protein was expressed and purified from *E. coli* lysates using IMAC, and CPD-mediated cleavage of the immobilized fusion protein was activated by InsP_6_ addition. As shown in Figure 4, autoprocessing occurred exclusively at the ICD-CPD interdomain junction, with a single protein equivalent to the size of His_6_-tagged ICD being released into the supernatant fraction. These results strongly suggest that the CPD will not promiscuously cleave target proteins.

### Fusion of Target Proteins to the CPD Can Increase Their Expression and Purity

**Figure 5 pone-0008119-g005:**
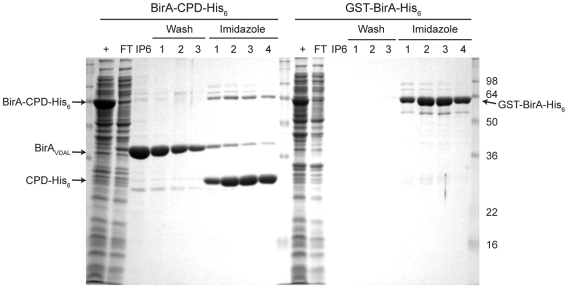
The CPD improves biotin ligase expression relative to a GST tag. SDS-PAGE analysis of purifications of biotin ligase (BirA, 35 kDa) fused to either CPD-His_6_ or GST-His_6_ using Coomassie stain. His_6_-tagged proteins bound to the Ni^2+^-NTA resin were incubated with 50 µM InsP_6_ for 1 hr at room temperature, and the resin was washed three times, followed by elution of Ni^2+^-bound proteins by 200 mM imidazole. CL, cleared lysate, +, IPTG induced culture, FT, flowthrough, IP6, elution from InsP_6_ incubation.

We noticed that the expression of the ICD-CPD-His_6_ fusion protein was at least three-fold higher than the ICD-His_6_ protein in *E. coli* lysates (Figure 4, compare + lanes). This result suggested that the CPD might generally enhance target protein expression and/or solubility levels. To test this hypothesis, we compared the expression and solubility of CPD fusions to several other target proteins carrying either a His_6_-tag and/or GST-fusion tag (Figures 5-8 and Table 1). In all cases, the presence of the CPD-His_6_ fusion tag increased the expression and solubility of target proteins. For example, fusion of the CPD-His_6_ tag to biotin ligase (BirA) from *E. coli* (BirA-CPD-His_6_) raised BirA expression levels by three-fold over the GST-BirA construct [13] (Figure 5 and Table 1).

**Table 1:** Target proteins expressed and purified by CPD purification method.

Target protein	Yield^a^ (mg/L culture)	Yield (nmol/L culture)	Activity
GFP_VDAL_ (CPD method)	3.3	105	Fluorescence at 511 nm
gp130(ICD)_VDAL_ (CPD method)	5.9	188	n/a
gp130(ICD)-His_6_	3.7	115	n/a
BirA_VDAL_ (CPD method)	10.9	202	Biotinylates LHHILDAQKMVWNHR BirA biotinylation site
GST-BirA-His_6_	12.0	90	Biotinylates LHHILDAQKMVWNHR BirA biotinylation site
PfSENP1_VDAL_ (CPD method)	2.0	67	Cleaves PfSUMO
_GSHM_PfSENP1	1.4	46	Cleaves PfSUMO
STIM1(CAD)_VDAL_	2.1	148	Binds Orai1
GST-STIM1(CAD)-His_6_	2.5^b^	62	n/a
MMP12_VDAL_ (CPD method)	1.4	47	Cleaves fluorogenic peptide substrate Mca-PLGLDL(Dpa)AR
MMP12 (refolded)	23	767	Cleaves fluorogenic peptide substrate Mca-PLGLDL(Dpa)AR

a Protein yield per litre of culture.

b Yield difficult to assess since GST-fusion protein degrades and falls out of solution over time.

**Figure 6 pone-0008119-g006:**
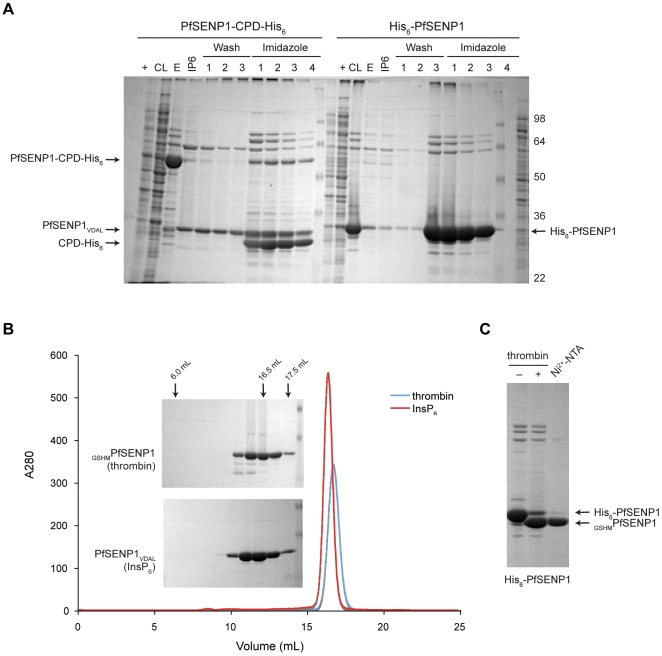
Comparison of His_6_-tag removal from *Plasmodium falciparum* SENP1 using thrombin relative to the CPD. (A) Coomassie stain of SDS-PAGE analysis of *P. falcipaurm* SENP1 (PfSENP1, 25 kDa) purified using either the CPD-His_6_ or His_6_-affinity tags. PfSENP1-CPD-His_6_ or His_6_-PfSENP1 bound to the Ni^2+^-NTA resin was incubated with 100 µM InsP_6_ for 2 hr at room temperature; the resin was washed three times, and wash fractions were collected. Ni^2+^-bound proteins were eluted by adding 200 mM imidazole. +, IPTG induced culture, CL, cleared lysate, E, imidazole elution prior to InsP_6_ addition, IP6, elution following InsP_6_ incubation. (B) UV trace PfSENP1 further purified by gel filtration chromatography following His_6_-tag removal. Inset, Coomassie stain of gel filtration fractions of PfSENP1 purifications. Thrombin refers to PfSENP1 purified following thrombin-mediated removal of the N-terminal His_6_-tag, while InsP_6_ refers to InsP_6_-induced, CPD-mediated removal of the C-terminal CPD-His_6_-tag. The residues added to the resulting PfSENP1 protein are shown: GSHM is added to the N-terminus of PfSENP1 following thrombin cleavage, while VDAL is added to the C-terminus of PfSENP1 following InsP_6_-activated CPD cleavage). (C) Coomassie stain of SDS-PAGE analyses of fractions taken during His_6_-PfSENP1 purification prior to thrombin incubation (–), following 12 hr thrombin incubation (+), and following subtractive IMAC to remove uncleaved His_6_-PfSENP1 (Ni^2+^-NTA). The yield of PfSENP1 diminished with each experimental manipulation.

The CPD purification system also enhanced the expression and purity of a previously uncharacterized SUMO/Sentrin-specific peptidase 1 (SENP1) from the parasitic pathogen *Plasmodium falciparum*, the causative agent of malaria (Figure 6) [14]. Although PfSENP1 carrying an N-terminal His_6_-tagged can be readily expressed and purified from *E. coli*, a number of contaminating bands are present, and the N-terminal His_6_-tag must be removed by the addition of thrombin followed by multiple chromatography steps (Table 2). In contrast, when PfSENP1 is expressed as a fusion to CPD-His_6_ and released as untagged PfSENP1 upon InsP_6_ addition, only one minor contaminant co-purifies with PfSENP1 (Figure 6B). This variant is easily removed using gel filtration chromatography (Figure 6C), and the untagged PfSENP1 is of sufficient purity that we have used it to obtain diffraction-quality crystals (E. Ponder, unpublished results). Notably, although the heterologous expression of *P. falciparum* proteins in *E. coli* is typically challenging [15], we have observed that this system can enhance the expression and purification of other parasite proteins from *P. falciparum* and a related apicomplexan parasite *Toxoplasma gondii*.

**Table 2:** Comparison of CPD-mediated and thrombin-mediated purification of PfSENP1.

	PfSENP1-CPD-His_6_	His_6_-PfSENP1
Step 1	Prepare soluble lysate (1 hr)	Prepare soluble lysate (1 hr)
Step 2	IMAC purification (2 hr)	IMAC purification (1 hr)
Step 3	On-bead cleavage; collect supernatant (2 hr)	Imidazole elution
Step 4	Concentrate protein (0.5 hr)	Buffer exchange and concentrate protein (0.5 hr)
Step 5	Gel filtration chromatography (1 hr)	Thrombin cleavage overnight (>12 hr)
Step 6	Concentrate protein (0.5 hr)	Remove His_6_-tag and uncleaved fusion with IMAC (1 hr)
Step 7		Concentrate protein and buffer exchange (0.5 hr)
Step 8		Gel filtration chromatography (1hr)
		Concentrate protein (0.5 hr)
**Total time**	5 hr	>17.5 hr

### Fusion of Target Proteins to the CPD Can Improve Their Stability and Solubility

**Figure 7 pone-0008119-g007:**
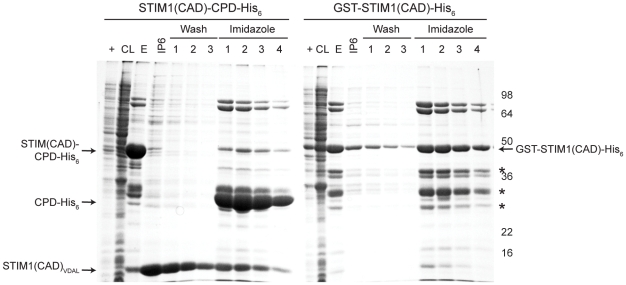
The CPD can improve the stability of target proteins. The Crac activation domain (CAD128) of STIM1 (amino acids 342–469, 14 kDa) was expressed in *E. coli* fused to either CPD-His_6_ or GST-His_6_. Asterisks indicate GST-STIM1(CAD)-His_6_ derived degradation products. His_6_-tagged proteins bound to the Ni^2+^-NTA resin were incubated with 50 µM InsP_6_ for 1 hr at room temperature, and the resin was washed three times, followed by elution of Ni^2+^-bound proteins by 200 mM imidazole. CL, cleared lysate, +, IPTG induced culture, FT, flowthrough, IP6, elution from InsP_6_ incubation.

In addition to augmenting the expression of target proteins, CPD-His_6_ fusions protected target proteins from proteolytic degradation. This effect was observed when the CRAC-activation domain (CAD) of the ER calcium sensor STIM1 was fused to the CPD (Figure 7). CAD is a small 107 aa polypeptide that activates Ca^2+^ release-activated Ca^2+^ (CRAC) channels by binding to the CRAC channel protein Orai1 [16]. Until now, large-scale expression and purification of this important regulatory domain has proven difficult due to its apparent instability even when fused to GST (Figure 7). However, using the CPD system, we were able to obtain significant quantities of a CAD-containing polypeptide (CAD128), which has subsequently been used in high-throughput screens for Orai1-CAD binding partners (A.M. Sadaghiani).

**Figure 8:**
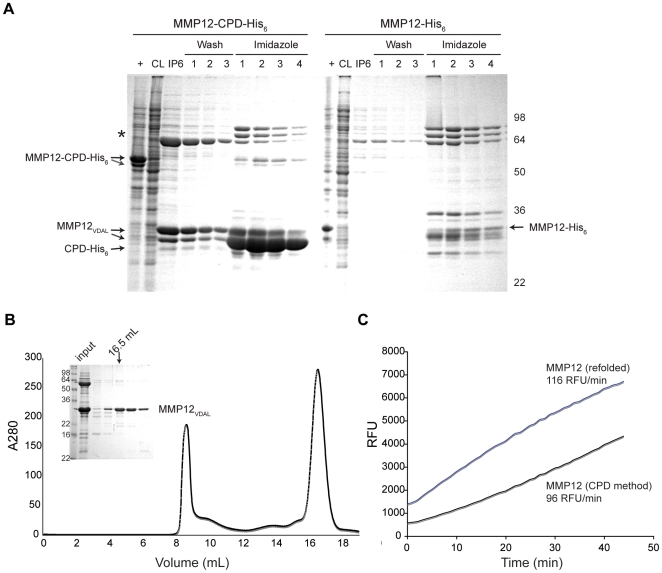
The CPD improves the solubility of mouse macrophage metalloelastin (MMP12) relative to a His_6_-affinity tag. (A) SDS-PAGE analysis of His_6_-tagged MMP12 using Coomassie stain. MMP12 was fused to His_6_-tagged CPD, and the expression of the fusion protein relative to His_6_-tagged MMP12 was compared. Asterisk indicates a putative chaperone protein that co-purifies with MMP12_VDAL_. The diagonal arrows indicate a His_6_-tagged truncated MMP12 product that is also observed during MMP12 purification from inclusion bodies [17]. His_6_-tagged proteins bound to the Ni^2+^-NTA resin were incubated with 50 µM InsP_6_ for 1 hr at room temperature, and the resin was washed three times, followed by elution of Ni^2+^-bound proteins by 200 mM imidazole. CL, cleared lysate, +, IPTG induced culture, FT, flowthrough, IP6, elution from InsP_6_ incubation, E, imidazole elution prior to InsP_6_ addition. (B) Additional purification of MMP12_VDAL_ by gel filtration chromatography. Inset, Coomassie stain of SDS-PAGE analysis of gel filtration fractions of MMP12_VDAL_. (C) MMP12 activity assay. The activity of MMP12 purified under denaturing conditions and refolded (MMP12 (Refolded)) and MMP12_VDAL_ purified using the CPD system (CPD method) against a standard fluorogenic substrate were compared. Comparable rates of fluorogenic substrate cleavage are observed for MMP12 purified by the CPD method relative to the refolding method.

Finally, the CPD purification system also increased the solubility of difficult-to-express proteins. Fusion of the mouse macrophage metalloelastase (MMP12) to CPD-His_6_ facilitated its purification from the soluble fraction of *E. coli* lysates, whereas His_6_-tagged MMP12 remained largely insoluble (Figure 8A). The currently used method for purification of His_6_-tagged MMP12 is a laborious procedure that requires solubilization of MMP12 inclusion bodies, refolding over multiple days, followed by anion and cation exchange chromatography [17]. The CPD purification system dramatically simplifies this purification procedure, allowing soluble, active MMP-12 to be isolated in approximately 7 hours (Figs. 8B and C, Table 3). We have used this improved purification protocol to rapidly express, purify and analyze MMP12 mutant proteins.

**Table 3:** Comparison of CPD method to published method for purifying matrix metalloelastin.

	MMP12-CPD-His_6_	MMP12-His_6_ [_17_]
Step 1	Prepare soluble lysate (1 hr)	Prepare and dissolve inclusion bodies (16 hr)
Step 2	IMAC purification (2 hr)	Protein refolding by dialysis in 1/20 volume (48 hr)
		a. 24 hr–6 M Urea, 4 L
		b. 12 hr–3 M Urea, 4 L
		c. 12 hr–1 M Urea, 4 L
Step 3	On-bead cleavage; collect supernatant (2 hr)	Load partially refolded protein on anion and cation exchange tandem columns (2.5 hr)
Step 4	Concentrate protein (0.5 hr)	Wash columns with three buffers to fully refold protein on column (2 hr)
Step 5	Gel filtration chromatography (1 hr)	Elute protein from cation exchange column (0.5 hr)
Step 6	Concentrate protein (0.5 hr)	Concentrate protein (0.5 hr)
**Total time**	7 hr	3–4 days

## Discussion

We have developed a novel one-step purification system that accelerates untagged recombinant protein purification from bacterial systems. By directly fusing an affinity-tagged, site-specific protease to a target protein, the CPD system ensures rapid and efficient removal of the fusion tag in a cost-effective manner. As a result, the CPD system overcomes many of the disadvantages associated with the exogenous addition of site-specific proteases, like thrombin and TEV protease, to remove fusion tags. These disadvantages can include their expense, generally low activity [2], [18], sensitivity to buffer conditions, and cleavage of target proteins at spurious sites [2]. In contrast, the CPD rapidly completes tag removal within two hours of addition (Figures 3-8), since the CPD is present at a 1∶1 ratio to the target protein and poised to undergo the autocleavage reaction [5]. Furthermore, the responsiveness of the protease specifically to InsP_6_ provides the user with complete control over the timing and conditions of fusion tag removal, while the autoprocessing nature of the CPD confers a high degree of specificity to fusion tag removal [4], [5]. Specifically, the protease is poised to undergo autocleavage upon InsP_6_ addition and exhibits poor transcleavage efficiency, as evidenced by the lack of CPD-mediated cleavage within any of the target proteins tested (Figures 3–8), including an intrinsically unstructured protein (Figure 4).

While purification systems based on fusing a protease to target proteins have previously been developed [9], [10], our demonstration that the CPD can enhance the expression, solubility, and stability of target proteins (Figures 4–8) suggests that the CPD system likely represents an improvement over existing methods like the intein-chitin-binding-domain (CBD) [9], [10] and sortase-His_6_ one-step purification systems [9]. Although these self-cleaving systems simplify the purification of well-expressed proteins, the large size of the intein-CBD fusion tag can decrease target protein solubility [2], while sortase-His_6_ fusion tags do not increase target protein solubility [9]. Furthermore, unlike self-cleaving elastin-like polypeptide (ELP) tags [19], CPD fusion proteins do not need to be subjected to temperature cycles, pH shifts, or high salt concentrations, a feature that is critical for the purification of intractable proteins. Based on the properties reported here, the CPD could replace the intein-tag in the self-cleaving-ELP system and potentially improve the solubility of ELP-tagged proteins while retaining their self-cleavability [19].

Indeed, a considerable strength of this method is that the CPD remains active over a wide range of conditions. CPD-mediated cleavage is complete within 1–2 hrs at temperatures between 4°C and 37°C, requires only micromolar concentrations of the small molecule InsP_6_ (an abundant and inexpensive reagent), and occurs efficiently both in the presence of standard protease inhibitor cocktails and in the absence of salt. This latter property carries the additional advantage of allowing the user to determine the buffer system in which to elute the target protein, eliminating the need for desalting or buffer exchange steps that can reduce protein yields. In addition, we have created a number of vector backbones that can be used to vary the residues that are appended to the target protein following CPD-mediated cleavage, which can range from a single amino acid residue to an HA epitope tag (Figure 2). Thus, the CPD system allows for considerable flexibility in optimizing purification procedures, as is often necessary for uncharacterized target proteins.

This versatility, combined with our observation that it can improve the solubility and integrity of difficult-to-express proteins (Figures 5 to [Fig pone-0008119-g006] [Fig pone-0008119-g007] 8), suggests that it will have widespread utility in biological research. The simplicity of this system will also make it amenable for large-scale proteomic, structural genomic, and commercial applications by eliminating the cost and complexity associated with exogenous site-specific proteases, potentially permitting its use in robotic systems for constructing protein arrays for screening purposes.

## Materials and Methods

### Bacterial Growth Condition

Overnight bacterial strains were grown at 37°C in Luria-Bertrani (LB) broth. Antibiotics were used at 100 µg/mL carbenicillin for pET22b vectors expressed in *E. coli*.

### Strain Construction

Primers used are listed in Table S1; strains constructed are listed in Table S2 in the Supporting Information. For construction of pET-CPD_SalI_ vectors, DNA encoding *Vibrio cholerae* MARTX toxin amino acids 3440-3650 from *Vibrio cholerae* N16961 was PCR amplified from genomic DNA using primers #1 and #2. The resulting PCR fragment was cloned into the SalI and XhoI sites of the pET22b and pET28a expression vectors, respectively (Novagen). For construction of the pET-CPD_SacI_ vector, DNA encoding *Vibrio cholerae* MARTX toxin amino acids 344**2**-3650 from *Vibrio cholerae* N16961 was PCR amplified from genomic DNA using primers #3 and #2, and the resulting PCR fragment was cloned into the SacI and XhoI sites of pET22b. To construct the pET-HA-CPD_SalI_ vectors, DNA encoding the HA epitope tag was added to the 5′ end of primer #4, and PCR amplification using primers #4 and #2 was used to fuse the HA tag directly to amino acid 3440 of *V. cholerae* MARTX CPD. The resulting PCR fragment was cloned into the SalI and XhoI sites of the pET22b and pET28a expression vectors, respectively. For construction of the pET-CPD_BamHI-Leu_ vector, DNA encoding *Vibrio cholerae* MARTX toxin amino acids 344**4**-3650 from *Vibrio cholerae* N16961 was PCR amplified from genomic DNA using primers #5 and #2, and the resulting PCR fragment was cloned into the BamHI and XhoI sites of pET22b. For construction of the pET-CPD_BamHI_ vector, DNA encoding *Vibrio cholerae* MARTX toxin amino acids 3440-3650 from *Vibrio cholerae* N16961 was PCR amplified from genomic DNA using primers #6 and #2, and the resulting PCR fragment was cloned into the BamHI and XhoI sites of pET22b.

The pET22b-GFP-CPD construct was cloned by PCR amplifying GFP from pEGFPN3 (Clontech) using primers #7 and #8. To construct the pET22b-gp130(ICD)-CPD vector, amino acids 642-918 of gp130 corresponding to the intracellular domain were PCR amplified using primers #9 and #10 and pET21a-gp130(ICD) as a template. The pET22b-BirA-CPD vector was constructed by PCR amplifying the *birA* gene from a pGEX4T1-BirA template using primers #9 and #10. The pET22b-STIM1(CAD)-CPD plasmid was constructed by PCR amplifying DNA encoding amino acids 342–469 of STIM1 using pGEX6-CAD128 as a template and primers #13 and #14. The pET22b-mMMP12-CPD construct was constructed by PCR amplifying the catalytic domain of mouse MMP12 (amino acids 29–267) using pET41a-mMMP12 as a template using primers #15 and #16. In all cases, the resulting PCR products were cloned into the NdeI and SalI sites of pET22b-CPD_SalI_.

### Protein Expression and Purification

For purification of His_6_-tagged CPD fusion proteins, overnight cultures of the appropriate strain were diluted 1∶500 into 1 L 2YT media and grown shaking at 37°C. When an OD_600_ of 0.6 was reached, IPTG was added to 250 µM, and cultures were grown for 3-4 hrs at 30°C. Cultures were pelleted, resuspended in 25 mL lysis buffer [500 mM NaCl, 50 mM Tris-HCl, pH 7.5, 15 mM imidazole, 10% glycerol] and flash frozen in liquid nitrogen. Lysates were thawed, then lysed by sonication and cleared by centrifugation at 15,000×g for 30 minutes. His_6_-tagged CPD fusion proteins were affinity purified by incubating the lysates in batch with 0.5–1.0 mL Ni-NTA Agarose beads (Qiagen) with shaking for 2–4 hrs at 4°C. The binding reaction was pelleted at 1,500×g, the supernatant was set aside, and the pelleted Ni^2+^-NTA agarose beads were washed three times with lysis buffer. In some cases, 10% of the Ni^2+^-NTA beads containing immobilized CPD-His_6_ fusion proteins were removed, pelleted and then His_6_-tagged fusion protein eluted using high imidazole buffer [500 mM NaCl, 50 mM Tris-HCl, pH 7.5, 175 mM imidazole, 10% glycerol].

To liberate untagged target proteins into the supernatant fractions, 300–500 µL lysis buffer was added to the Ni^2+^-NTA beads containing CPD-His_6_ fusion proteins and the indicated amount of inositol hexakisphosphate (InsP_6_, Calbiochem) was added. In general, on-bead cleavage was allowed to proceed by nutating the beads in the presence of 50–100 µM InsP_6_ for 1–2 hr at either room temperature or 4°C. The beads were pelleted at 1,500×g, and the supernatant fraction was removed. The beads were then washed 3–4 times with 300–500 µL lysis buffer, and supernatant fractions retained. His_6_-tagged proteins remaining on the beads (i.e. cleaved CPD-His_6_) were eluted using high imidazole buffer [500 mM NaCl, 50 mM Tris-HCl, pH 7.5, 175 mM imidazole, 10% glycerol] in 300–500 µL volumes. The elution was repeated 3–4 times, and eluate fractions were collected. Purification of His_6_-tagged proteins lacking the CPD was performed in parallel.

This general procedure was followed with the following exceptions: for purification of MMP12 constructs, the cultures were grown at 16°C for 8 hr after IPTG induction, and 1 mM tris(2-carboxyethyl)phosphine (TCEP) was added to the lysis buffer to prevent misfolding of the protein. PfSENP1 and BirA protein purifications were performed exclusively at room temperature, since at 4°C, protein aggregation was observed. For removal of the His_6_-tag from His_6_-PfSENP1, thrombin beads (Calbiochem) that had been washed in PBS were added to the eluted His_6_-PfSENP1, which had been buffer exchanged into PBS according to the manufacturer's instructions. Thrombin cleavage was allowed to proceed with shaking overnight for 12 hr at room temperature. Aliquots were taken before and after thrombin addition to monitor cleavage efficiency. Thrombin cleaved, untagged PfSENP1 was enriched by performing a subtractive Ni^2+^-NTA pull-down. Untagged PfSENP1 from both methods was then buffer-exchanged into gel filtration buffer (50 mM NaCl, 20 mM Tris pH 8.0). Protein purifications were analyzed by SDS-PAGE and Coomassie staining using GelCode Blue (Pierce). Purified protein concentrations of purified were determined by Bradford assay (Pierce).

### Purification of MMP12-His_6_

MMP12-His_6_ was purified as previously described [17] with the following modifications. The cell pellet was resuspended in 100 mM NaCl, 100 mM Tris pH 8.0, 5.0 mM EDTA, 0.5 mM DTT, 100 µg/mL lysozyme and stirred for 2 hr. The cells were sonicated then centrifuged at 10,000 rpm for 10 min. The resulting inclusion bodies were washed two times and then resuspended in 50 mL 6M guanidine hydrochloride, 10 mM Tris pH 8.0 by stirring at 4°C overnight. The mixture was centrifuged at 15,000 rpm for 30 min, and 2 mL aliquots of supernatant were prepared. The supernatant was diluted 1∶100 into denaturing buffer [6M Urea, 50 mM Tris pH 8.0, 10 mM CaCl2, 30 mM NaCl, 5 mM DTT] to a final concentration of 0.1–0.2 mg/mL. The protein was then dialyzed for 24 hr in 2 L refolding buffer 1 [3 M Urea, 50 mM Tris pH 8.0, 10 mM CaCl_2_, 30 mM NaCl, 5 mM DTT]. The partially refolded protein was then dialyzed in 4 L of refolding buffer 2 [1 M Urea, 50 mM HEPES pH 7.4, 10 mM CaCl_2_, 5 mM DTT). The buffer exchanged protein was then purified using tandem 5 mL MonoQ and SP Sepharose (GE Healthcare) at 4°C. After loading the protein on the column, the column was washed with 50 mL of refolding buffer 2 without DTT at 1 M, 0.5, and 0 M urea, respectively. The protein was eluted from the SP column in 500 mM NaCl, 50 mM HEPES pH 7.4, 10 mM CaCl_2_.

### Gel Filtration Chromatography

Untagged PfSENP1 obtained from either thrombin or InsP_6_-mediated cleavage was concentrated using a 10 kDa Centricon concentrator (Millipore) and buffer exchanged into 50 mM NaCl, 20 mM Tris pH 8.0 and purified on a Superdex 200 10/30 column (GE Healthcare) equilibrated in the same buffer. For MMP12, the gel filtration buffer contained 150 mM NaCl, 50 mM Tris pH 7.4, 10 mM TCEP. Gel filtrations were performed at 4°C.

### Activity Assays

Fluorescence of purified GFP at 511 nm was verified using a Molecular Devices fmax plate reader in black 96-well plates and 488 nm excitation. The activity of MMP12 was determined using the fluorogenic substrate Mca-PLGLDL(Dpa)AR (Mca, (7-methoxycoumarin-4-yl)acetyl, Dpa, *N*-3-(2,4-dinitrophenyl)-L-2,3-diaminopropionyl, Anaspec). Reactions were performed in the assay buffer (50 mM Tris pH 7.4, 150 mM NaCl, 10 mM CaCl_2_, 0,02% NaN_3_, 5 mM TCEP) at 37°C. The substrate was used at 10 µM and the protein at 0.2 µM. The substrate hydrolysis was monitored continuously in a fluorescent plate reader (Molecular Devices) using an excitation wavelength of 325 nm and an emission wavelength of 395 nm.

## Supporting Information

Table S1 Primers used in Study. ^a^ Restriction enzyme sequences are underlined, and the HA tag is shown in italics. ^b^ RE - Restriction site
(0.06 MB DOC)

Table S2 Strains used in study. 1. Skiniotis G and Lupardus, PJ, *et al.* (2008) Mol Cell 31: 737–748. 2. Ponder EL, *et al.* (2009) under review Nat Chem Biol. 3. Park CY, *et al.* (2009) Cell 136: 876–890.
(0.07 MB DOC)
