# Supplementary material for: Simplified, Enhanced Protein Purification Using an Inducible, Autoprocessing Enzyme Tag
Source: PLoS One. 2009 Dec 2;4(12):e8119. doi: 10.1371/journal.pone.0008119 (PMC2780291; doi:10.1371/journal.pone.0008119)
Supplement: Table S1 — Primers used in Study. a Restriction enzyme sequences are underlined, and the HA tag is shown in italics. b RE - Restriction site (0.06 MB DOC) [file pone.0008119.s001.doc]

**Table S1. Primers used in study.**

| # | Name | Sequence^a^ | *RE*^b^ |
| --- | --- | --- | --- |
| 1 | 5’ SalI ∆50CPD | CACGTCGACGCATTAGCGGATGGAAAAATACTCCAT | *SalI* |
| 2 | 3’ XhoI CPD | CCGCTCGAGACCTTGCGCGTCCCAGCTTAG | *XhoI* |
| 3 | 5’ SacI CPD | TTCGAGCTCGCGGATGGAAAAATACTC | *SacI* |
| 4 | 5’ SalI HA-CPD | TCCGTCGAC*TACCCGTACGACGTCCCGGACTACGCG*GCATTAGCGGATGGAAAA | *SalI* |
| 5 | 5’ BamHI CPD_Leu_ | TCGGGATCCGGAAAAATACTCCATAATCAA | *BamHI* |
| 6 | 5’ BamHI CPD | TCGGGATCCGCATTAGCGGATGGAAAAATACTCCAT | *BamHI* |
| 7 | 5’ NdeI gfp | ATTCATATGGTGAGCAAGGGCGAG | *NdeI* |
| 8 | 3’ SalI gfp | CACGTCGACCTTGTACAGCTCGTCCAT | *SalI* |
| 9 | 5’ NdeI gp130(ICD) | ATTCATATGAATAAGCGAGACCTA | *NdeI* |
| 10 | 3’ SalI gp130(ICD) | TCCGTCGACCTGAGGCATGTAGCCGCC | *SalI* |
| 11 | 5’ NdeI BirA | GGCCATATGATGAAGGATAACACCGTGCCA | *NdeI* |
| 12 | 3’ SalI BirA | TCCGTCGACTTTTTCTGCACTACGCAGGGA | *SalI* |
| 13 | 5’ NdeI CAD | AGCCATATGTATGCTCCAGAGGCCCTT | *NdeI* |
| 14 | 3’ SalI CAD | TCCGTCGACAGCAGGGTTGGGGCGTGT | *SalI* |
| 15 | 5’ NdeI MMP12 | CTTCCATATGGCTCCCATG | *NdeI* |
| 16 | 3’ SalI MMP12 | TAACGTCGACCTCGAGTCC | *SalI* |
