# Supplementary material for: Simplified, Enhanced Protein Purification Using an Inducible, Autoprocessing Enzyme Tag
Source: PLoS One. 2009 Dec 2;4(12):e8119. doi: 10.1371/journal.pone.0008119 (PMC2780291; doi:10.1371/journal.pone.0008119)
Supplement: Table S2 — Strains used in study. 1. Skiniotis G and Lupardus, PJ, et al. (2008) Mol Cell 31: 737–748. 2. Ponder EL, et al. (2009) under review Nat Chem Biol. 3. Park CY, et al. (2009) Cell 136: 876–890. (0.07 MB DOC) [file pone.0008119.s002.doc]

**Table S2: Strains used in study**

| **Strain** | **Genotype and relevant features** | Reference |
| --- | --- | --- |
|  | BL21(DE3) | Novagen |
| 41 | DH5α | D.E. Cameron |
| 7 | pET22b in DH5α | D.E. Higgins |
| 269 | pET28a in DH5α | E. Ponder |
| 195 | pET22b-CPD_SalI_ | This study |
| 330 | pET28a-CPD_SalI_ | This study |
| 329 | pET22b-HA-CPD_SalI_ | This study |
| 331 | pET28a-HA-CPD_SalI_ | This study |
| 373 | pET22b-CPD_SacI_ | This study |
| 374 | pET22b-CPD_BamHI-Leu_ | This study |
| 375 | pET22b-CPD_BamHI_ | This study |
| 197 | pET22b-GFP-CPD­ | This study |
| 371 | pET22b-gp130(ICD)-CPD | This study |
| 372 | pET21a-gp130(ICD) | [1] |
| 228 | pET22b-BirA-CPD | This study |
| 183 | pGEX4T1-BirA | P.J. Lupardus |
| 360 | pET22b-PfSENP1-CPD | [2] |
| 361 | pET28a-PfSENP1 | [2] |
| 324 | pET22b-STIM1(CAD)-CPD | This study |
| 327 | pGEX6-CAD128 | [3] |
| 359 | pET22b-mMMP12 | This study |
| 358 | pET41a-mMMP12 | C. Overall |
